# The complete mitochondrial genomes of *Parabotia lijiangensis* (Cypriniformes: Botiidae)

**DOI:** 10.1080/23802359.2020.1771227

**Published:** 2020-06-01

**Authors:** Yujie Feng, Genxuan Wang

**Affiliations:** College of Life Sciences, Zhejiang University, Hangzhou, China

**Keywords:** *Parabotia lijiangensis*, mitochondrial genome, phylogeny, Cobitidae

## Abstract

In this study, we obtained the 16,579 base pair (bp) mitochondrial DNA sequence of *Parabotia lijiangensis*. The mitogenome encodes 13 protein-coding genes, 22 tRNA genes, 2 rRNA genes, a control region, and has a nucleotide composition of A: 30.8%, T: 25.2%, G: 16.1%, and C: 27.9% (AT content: 56.0%). The complete mitogenome of *P. lijiangensis* provides essential and important DNA molecular data for further phylogenetic and evolutionary analysis of the Botiidae family.

*Parabotia lijiangensis* (Cypriniformes, Botiidae, *Parabotia*), is a small-sized benthopelagic fish endemic to China, mainly distributed in the Lijiang river. It is hard to tell *P. lijiangensis* apart from *P. fasciata*, for these two species share the dark caudal spot and beautiful body stripe patterns (Jingxing 1980). Here, we first determined the complete mitochondrial genome of *P. lijiangensis* and reconstructed the phylogenetic relationship with other Botiidae species.

In this study, the sample of the *P. lijiangensis* was obtained from the Lijiang River (110°25′35.13″E, 25°10′50.23″N), Guangxi, China. The voucher specimen was deposited in Laboratory 121, College of Life Sciences, Zhejiang University, with identifier F1921120. Genomic DNA was extracted from muscle by using TIANamp Genomic DNA Kit following the manufacturer’s instructions (Tiangen Inc., Beijing, China).

DNA library preparation and 150-bp paired-end sequencing were performed on the Illumina HiSeq platform. After filtering, the mitochondrial data were assembled in NOVOPlasty version 2.63 (Dierckxsens and Mardulyn 2017), with *Parabotia fasciata* (KM393223) (Wei et al. 2014) as the seed reference. The mitochondrial genome of *P. lijiangensis* was 16,579 bp in length (GenBank with the accession number of MT323118) and share the same organization like other fish, consisting of 13 protein-coding genes (PCGs), 22 transfer RNA (tRNA) genes, 2 ribosomal RNA (rRNA) genes, and the control region.

NADH dehydrogenase subunit 6 (ND6) and eight tRNA genes (*Gln, Ala, Asn, Cys, Tyr, Ser, Glu* and *Pro*) are encoded on the light strand (L-strand), the remaining genes are located on the heavy strand (H-strand). Nucleotide base composition of the complete sequences was A: 30.8%, T: 25.2%, G: 16.1%, and C: 27.9%, thus the overall AT content was 56.0%. Among all 13 protein-coding genes, we found that most protein-coding genes for *P. lijiangensis* share the common initiation codon ATG, the exception being the COXI gene, which starts with GTG. Besides, incomplete termination codons (T or TA) were also found in six genes (COXII, COXIII, ND3, ND4, and Cytb).

Phylogenetic relationships of Botiidae were reconstructed using maximum likelihood (ML) method based on the multiple alignment of 15 mitochondrial genomes within this family and one outgroup *Cobitis takatsuensis.* ML analysis was conducted using RAxML-HPC version 8.2.8 with 1000 bootstrap replicates on the CIPRES Science Gateway website (Miller et al. 2010). The phylogenetic tree strongly supported the close relationship of *P. fasciatus*, *P. banarescui* and *P. lijiangensis*. And the former two species formed a clade sister to *P*. *lijiangensis* (Figure 1), which was also congruent with the previous studies (Slechtová et al. 2006; Tang et al. 2006).

**Figure 1.**
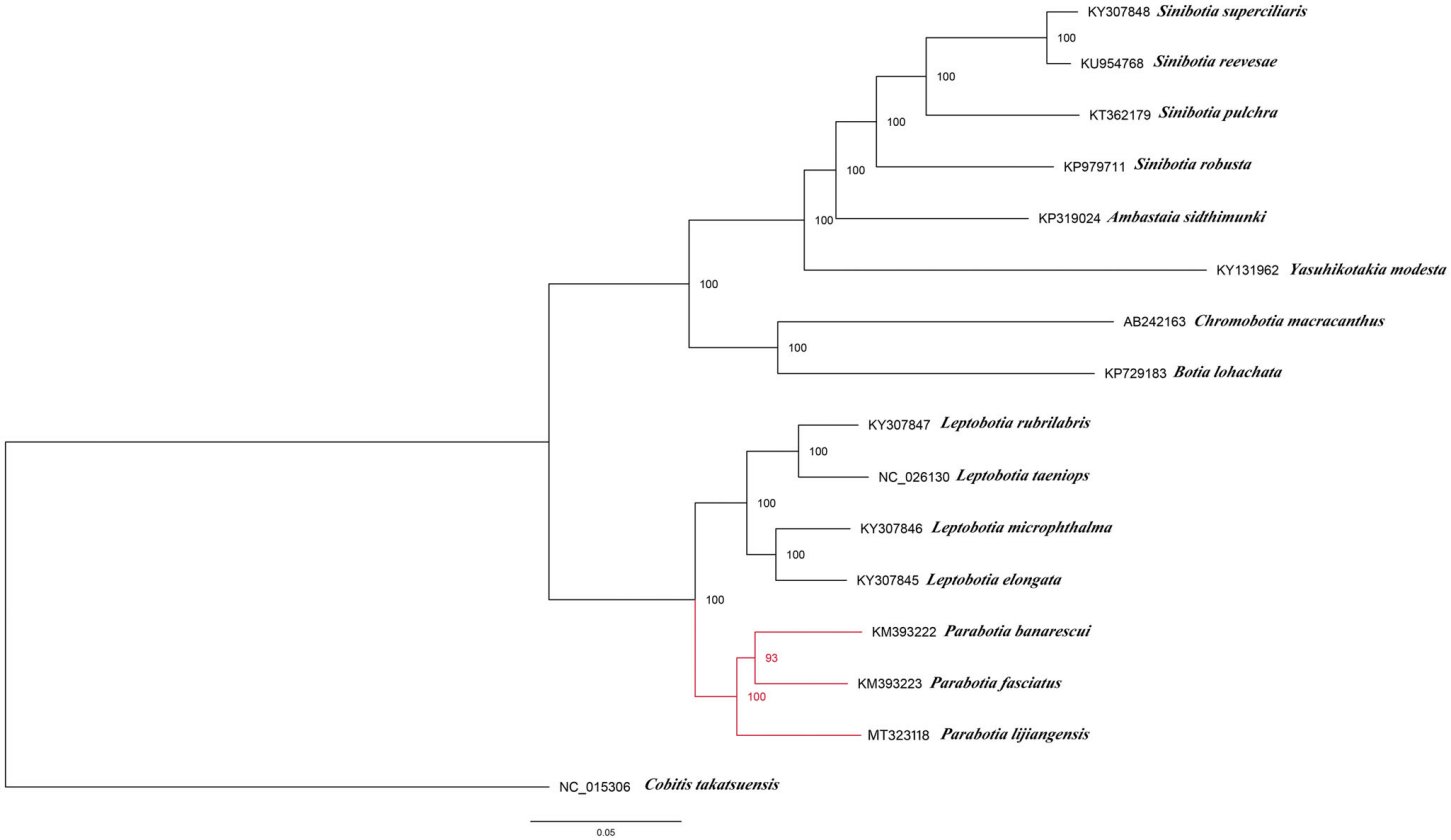
Phylogenetic tree using maximum-likelihood (ML) based on complete mitochondrial genome data of 15 Botiidae species with *Cobitis takatsuensis* as an outgroup. Numbers near the nodes represent ML bootstrap values. The GenBank accession number is listed next to each species within the tree.

## Data Availability

The data that support the findings of this study are openly available in Genbank with the accession codes MT323118 (https://www.ncbi.nlm.nih.gov/nuccore/MT323118).
